# Supplementary material for: Versican promotes T helper 17 cytotoxic inflammation and impedes oligodendrocyte precursor cell remyelination
Source: Nat Commun. 2022 May 4;13:2445. doi: 10.1038/s41467-022-30032-0 (PMC9068758; doi:10.1038/s41467-022-30032-0)
Supplement: Supplementary file 1 — Supplementary Information [file 41467_2022_30032_MOESM1_ESM.pdf]

Supplementary Table S1. Some of the characteristics of people with multiple sclerosis.

The data of these patients are presented in Figure 1C-E.

NHB Number	Sex	Age (y)	Disease Duration (y)	Lesion Type	Brain Region
2012-078	Male	58	30	Inactive WML	Inferior parietal lobe
2013-019	Female	53	16	Active WML	Superior frontal gyrus
2013-047	Female	48	22	Inactive WML	Inferior frontal gyrus
2014-007	Female	51	23	Inactive WML	Temporal pole
2014-038	Female	35	10	Chronic active WML	Superior frontal gyrus
2015-006	Female	57	28	Chronic active WML	Inferior parietal lobe

Supplementary Figure S1. **Additional analysis of MS brains.**

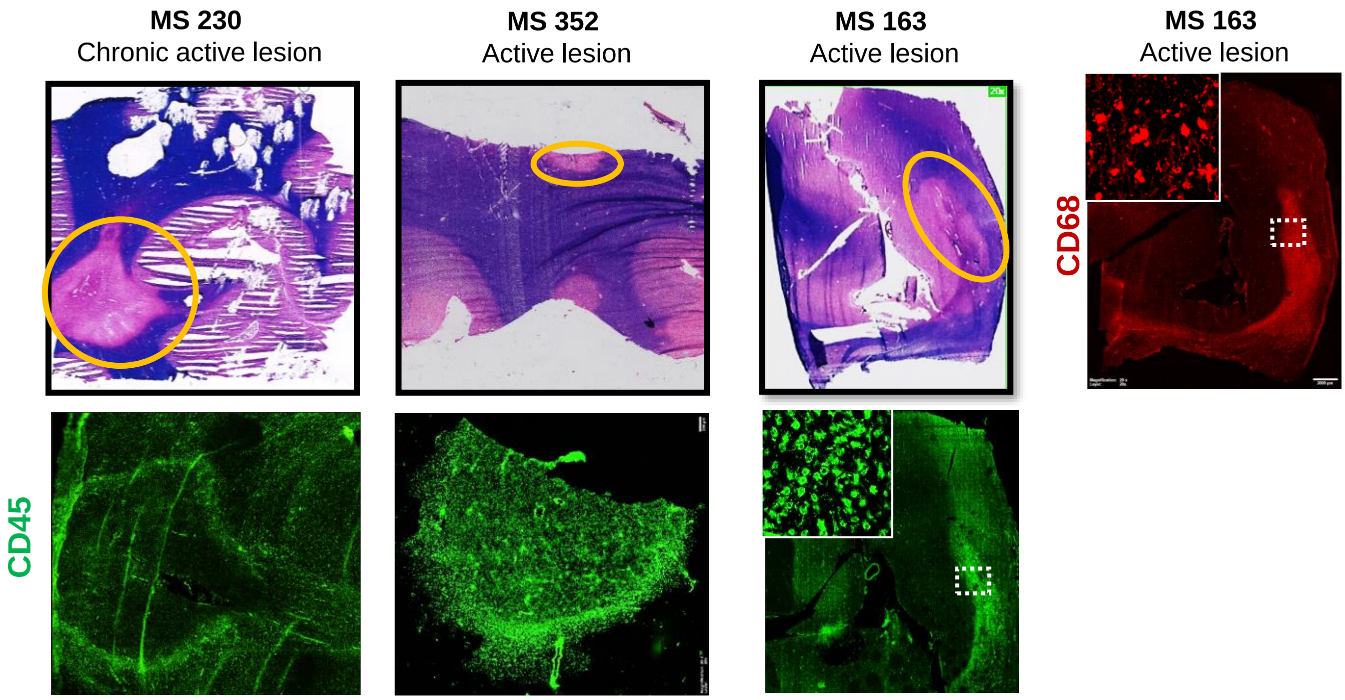

Frozen brain autopsy sections from three people with MS (MS163, 230, 352) were stained with LFB (top panel) to delineate demyelinated lesions by loss of LFB myelin staining. Tissues were labelled for CD45 (green) or CD68 (red) for immune cells. The lesion type was characterized as active (MS-163, MS-352) or chronic active (MS-230) by prominent accumulation of immune cells within the lesion or at the lesion rim, respectively. Images were acquired with slide scanner. The experiment was repeated twice. Yellow rings in top panel indicate lesions which are shown at higher magnification in the bottom panel.

Supplementary Figure S2. Spatial RNA sequencing of MS lesions reveals an inverse correlation between BCAS and versican mRNA transcripts.

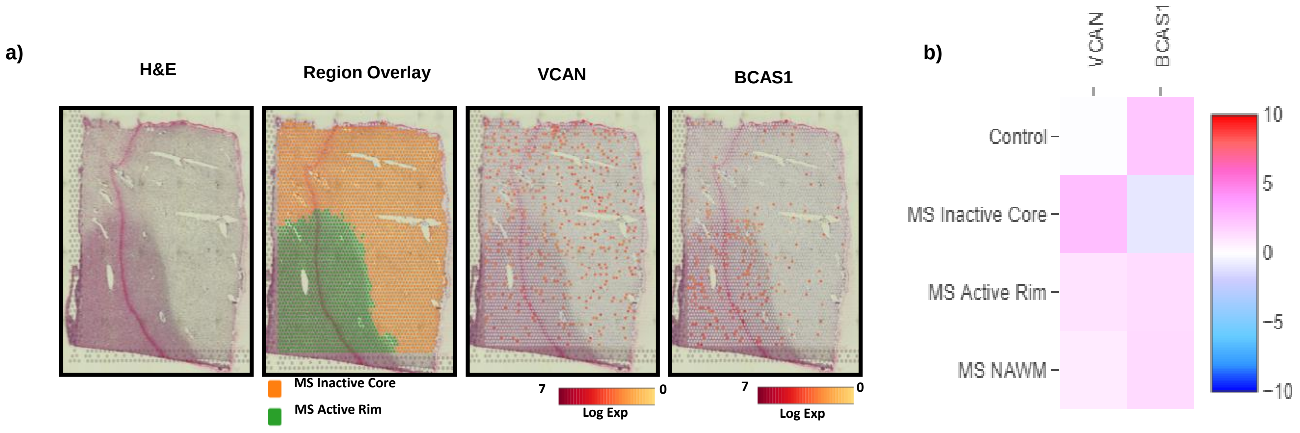

A) Representative images showing the histological demarcation and spatial RNAseq expression of versican (VCAN) and BCAS1 mRNA from a post-mortem MS brain specimen with active rim and the inactive core using H&E staining overlaid with spatially sequenced regions. B) Heatmap from the spRNAseq analysis of post-mortem MS brain tissue sections comparing the expression of VCAN and BCAS1 transcripts in the non-MS control and MS NAWM, active rim, and inactive core of chronic active lesions. The heatmap was generated using 2 healthy control brain samples and 2 MS specimens.

Supplementary Figure S3. Additional murine OPC culture experiments.

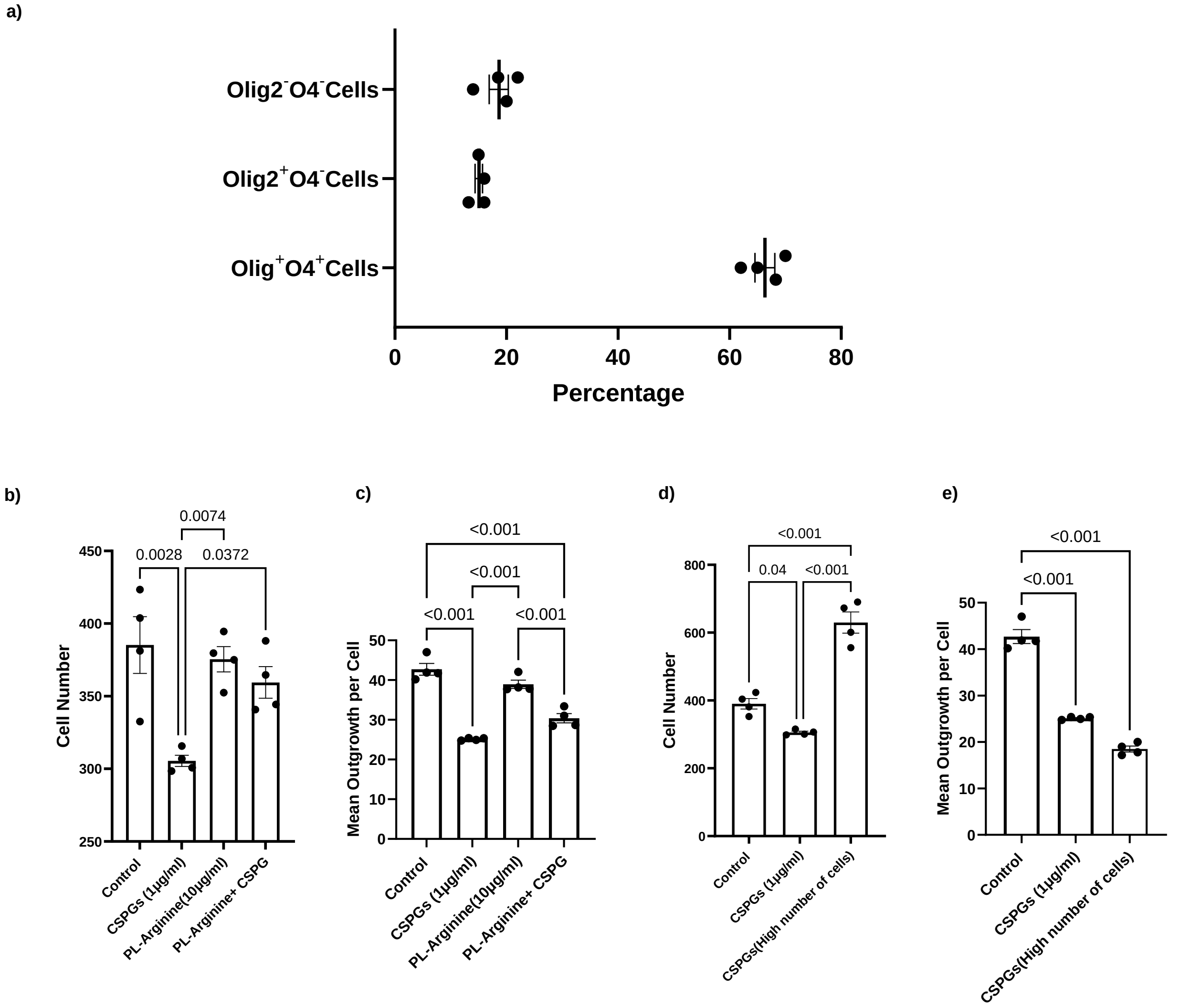

A) The proportion of cells expressing Olig2 (a transcription factor that marks all cells of the oligodendrocyte lineage) and O4 (a sulfatide which describes a stage of differentiation in the oligodendrocyte lineage) in a representative mouse OPC culture after 24h. More than 80% of cells are olig2^{+}. Each black circle is a separate well. B, C) Poly-arginine peptide (PL-Arginine) was added to CSPG substrate to counteract the negative charges of CSPGs. Number of cells (B) and mean process outgrowth (C) after 24h are shown. D, E) Bar graphs comparing the cell number (D) and mean process outgrowth (E) of mouse OPCs when 30,000 cells (3 times more than normal condition) were seeded on control or CSPGs after 24h. Data are presented as mean ± SEM. n=4 replicate wells of one representative experiment. Experiments were repeated twice. One-way ANOVA-Tukey’s post hoc Source data are provided as a source data file.

Supplementary Figure S4. CSPGs do not directly affect Th17 cell survival, proliferation, and activation.

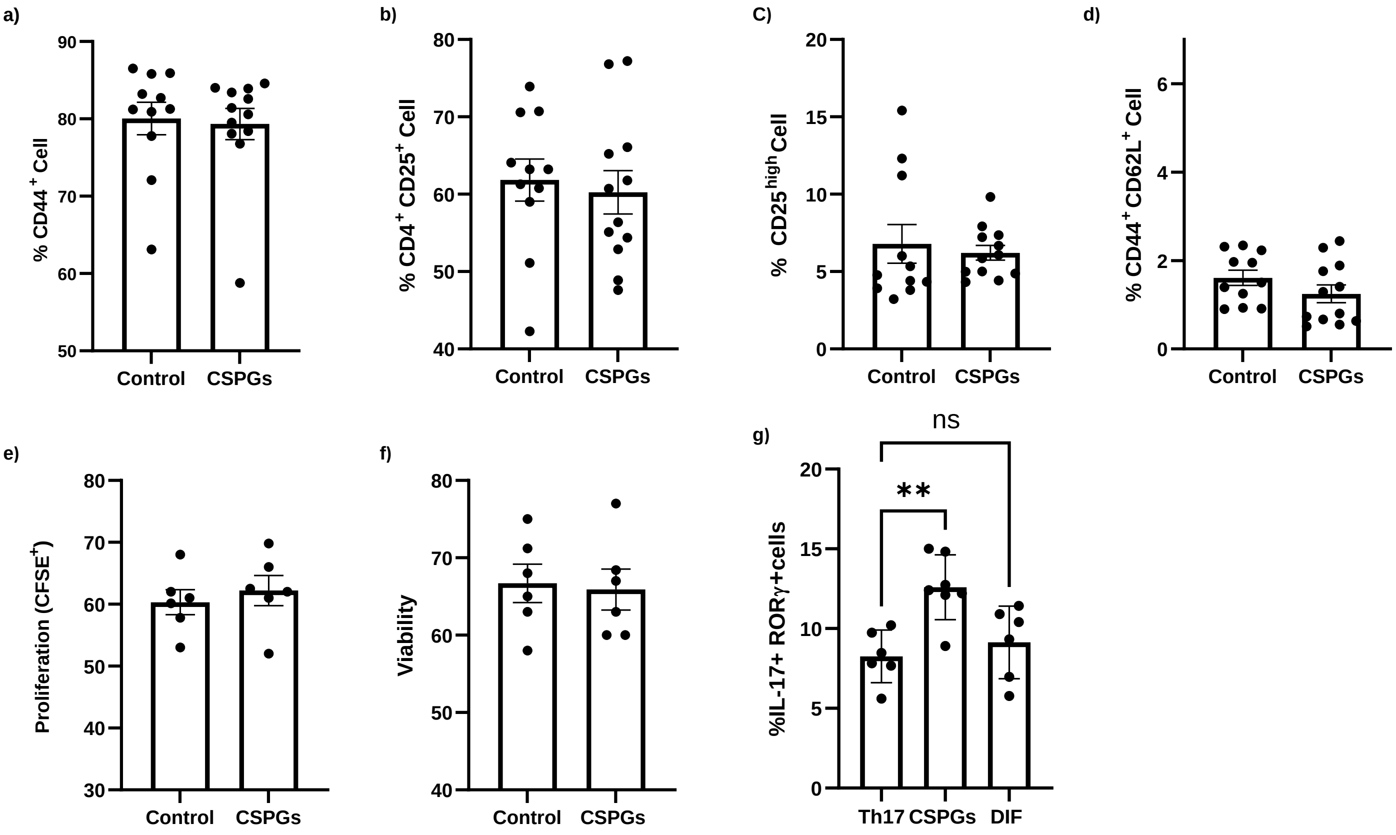

Naïve CD4⁺ T cells were cultured on CSPG-coated wells and were then activated and polarized to Th17 subset. While T cell activation markers such as CD44 and CD25 are upregulated following engagement of the T cell receptor, the expression of markers such as CD62L decreases. Percentage of positive cells for activation markers including A) CD44, B, C) CD25 and D) CD62L was determined using the flow cytometry 4 days later in the absence (control) or presence of CSPGs. Data are presented as mean ± SEM, n=11 replicates for control group, n=12 replicates for CSPGs group over three separate experiments. Two-tailed unpaired Student's t test. E) T cell proliferation was measured by CFSE dilution method. F) Cell survival was assessed using the viability stain which cannot pass through the impermeable plasma membranes of living cells. G) To exclude the possible effect of DIF on Th17 polarization, DIF (50 μM) was added to naïve CD4⁺ T cells which were then activated and polarized to Th17 subset. Bar graph compares the frequency of IL-17⁺RORγ⁺ Th17 cells. For E-G, data are presented as mean ± SEM, n=6 replicate wells from two separate experiments. Two-tailed unpaired Student's t test for E,F; one-way ANOVA- Bonferroni post hoc for G, significance indicated as **$p < 0.0024$. Source data are provided as a source data file.

Supplementary Figure S5. The effect of Treg cells on OPCs does not change after CSPG exposure.

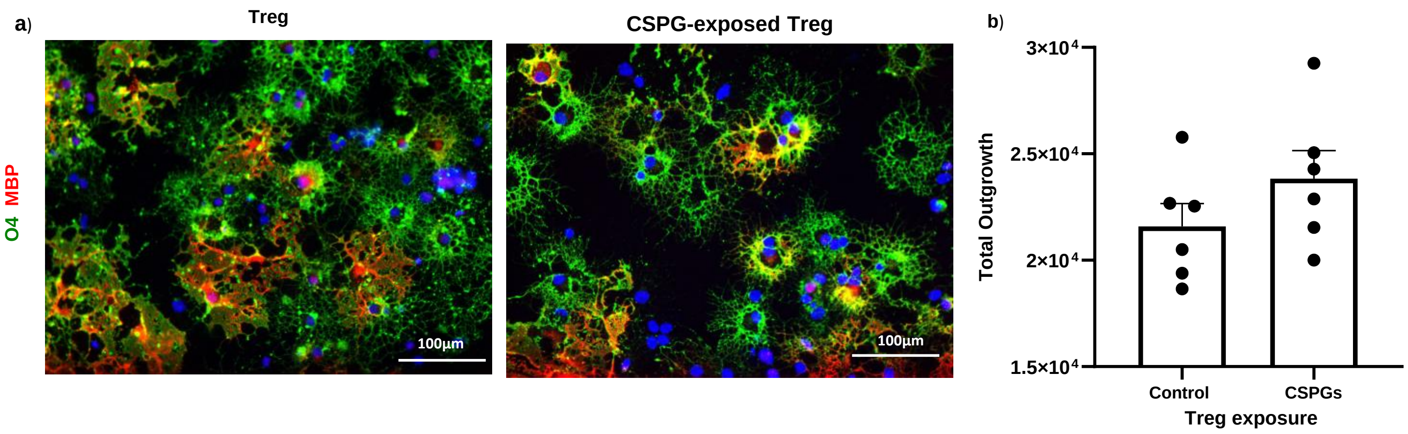

Treg cells or CSPG-exposed Treg cells were added to OPCs for 24h. Cells were then stained for O4 (green) and MBP (red) markers and OPC cell process outgrowth was measured using ImageXpress. A) Representative images from OPC co-culture with PBS- or CSPG-exposed Treg cells. B) Bar graph comparing OPC process outgrowth between PBS- or CSPG-exposed Treg after 24. Data are presented as mean ± SEM, n=6 replicate wells over two separate experiments experiment, two-tailed unpaired Student's t test. Source data are provided as a source data file.

Supplementary Figure S6. The frequency, maturation state and cytokine secretion of mature dendritic cells remain unaffected following CSPG exposure.

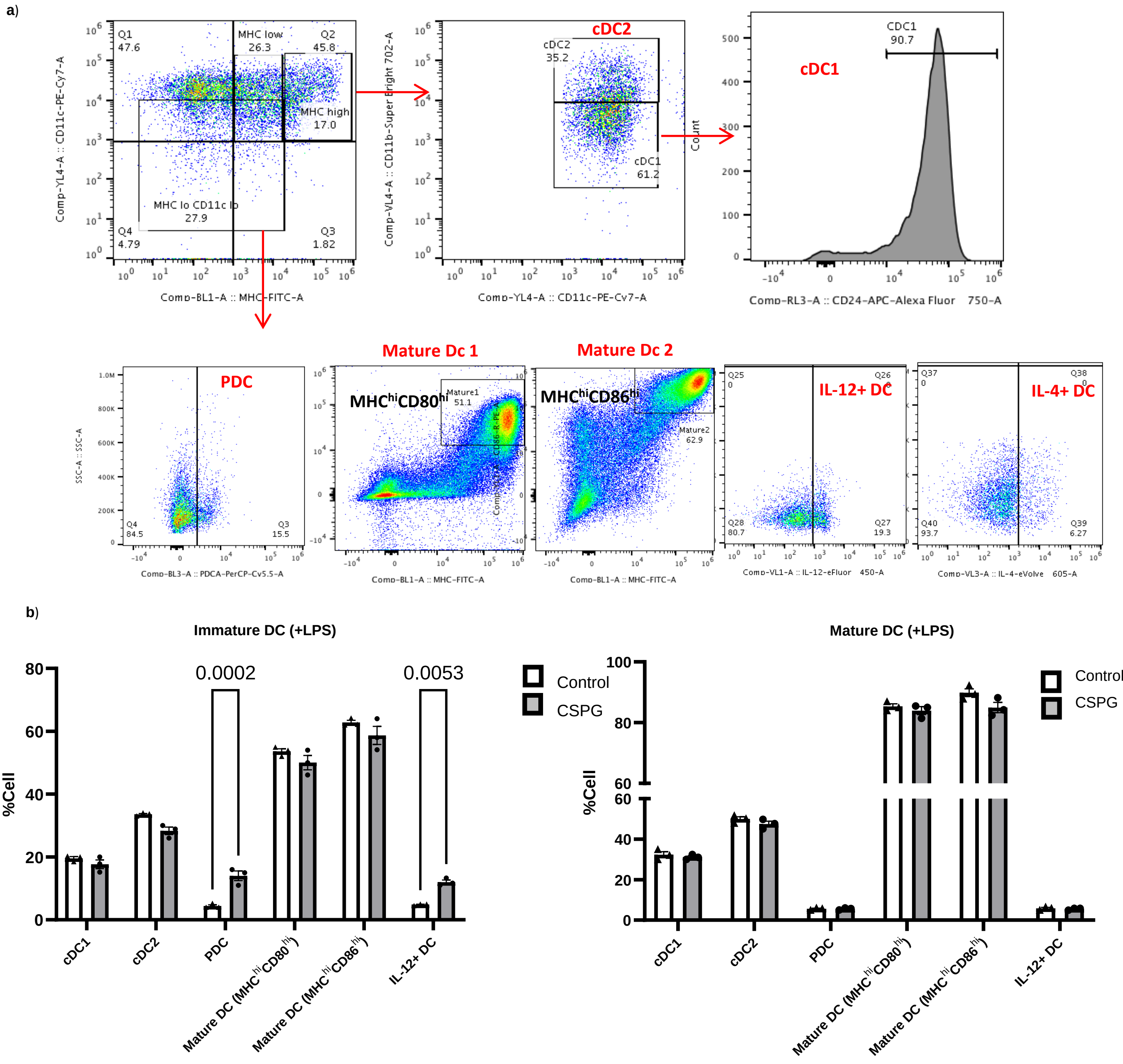

Bone marrow-derived dendritic cells (BMDCs) were seeded in control or CSPG-coated (10 µg/ml) 24-well plates at a density of 1×10^6 cells and treated with 50 µg/ml MOG in absence or presence of LPS (10 ng/ml) for 12 h. Different types of dendritic cells (plasmacytoid (PDC) and conventional dendritic cells (CDC1/2)) and maturation markers were analyzed by flow cytometry using anti mouse CD11b, CD11c, CD80, CD86, MHCII, PDCA-1, CD24 and IL-12. A) Gating strategies and B) bar graphs comparing the percentage of different DC subsets, mature DCs and IL-12$^+$ DCs between control and CSPG-treated wells in the absence (immature DC) or presence of LPS (mature DC). Data are presented as mean ± SEM, n=3 replicate wells from one experiment, two-way ANOVA with Sidak’s post-hoc test . Source data are provided as a source data file.

Supplementary Figure S7. Corroboration of transgenic NG2^{CreER}:MAPT^{mGFP} mouse as a suitable model to study remyelination in EAE.

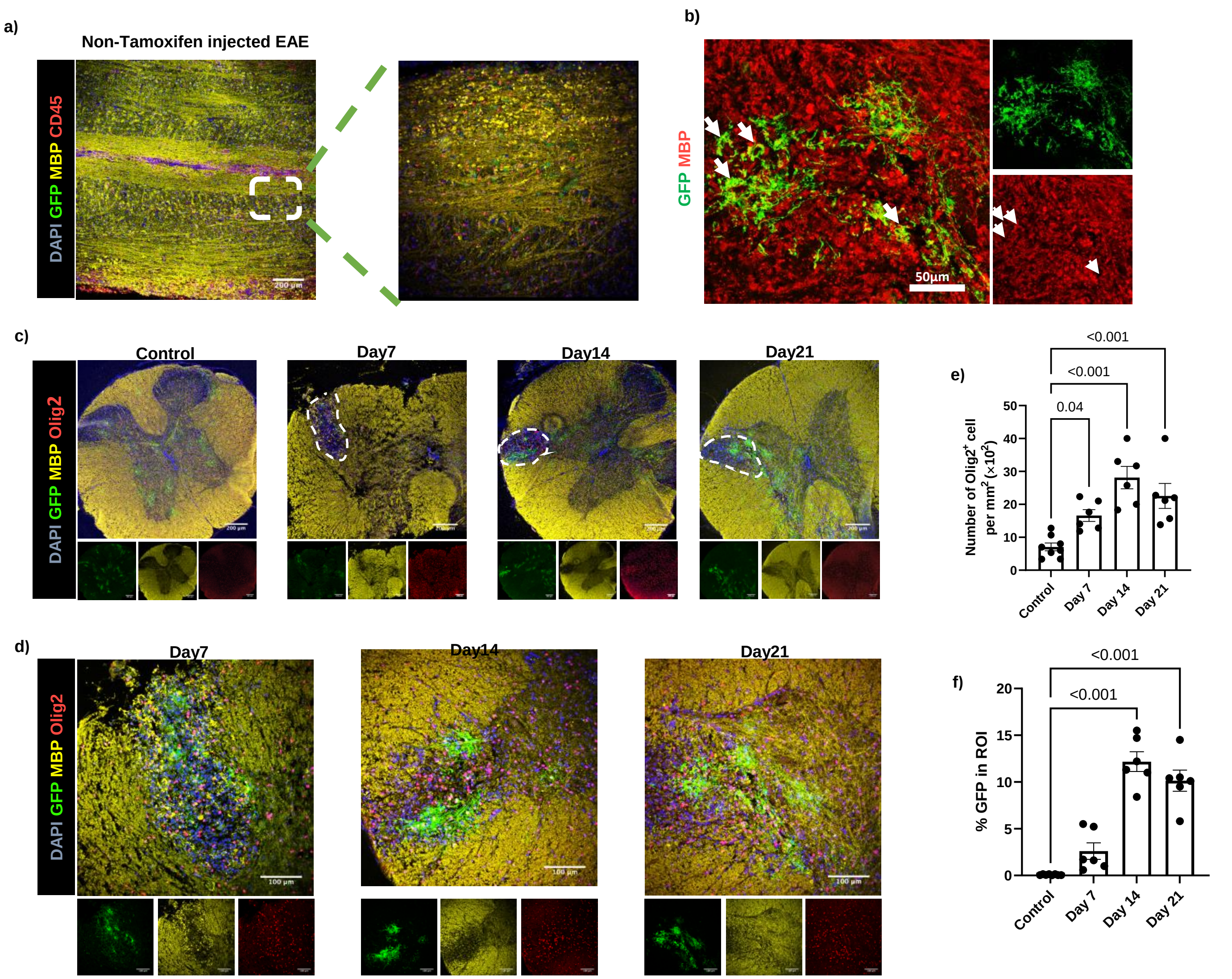

Tamoxifen-inducible Cre recombinase activity in NG2^{CreER}:MAPT^{mGFP} mice results in deletion of the floxed sequence leading to GFP expression in newly formed oligodendrocytes. A) To assess the Cre leakiness in the absence of tamoxifen, GFP expression was measured in EAE mice without tamoxifen injection. B) Tamoxifen injected mice were labeled for both GFP^{+} (green) and MBP (red) to show their correspondence. Arrow shows cell soma. Experiments were repeated twice. C) To corroborate NG2^{CreER}:MAPT^{mGFP} mice as a suitable approach to study remyelination, we used the lysolecithin model. Coronal sections of the lesion site were immunostained with GFP for newly formed oligodendrocytes (green), MBP for myelin (yellow) and olig2 for oligodendrocyte lineage cells (red). Representative images of low magnification images acquired by 10x objectives of immunofluorescent laser confocal microscope (Z-stack) are shown. Dotted lines indicate the lesion areas magnified in D (25x). E) Bar graphs compare the number of oligodendrocytes and F) percent GFP^{+} area within the lesion over the time post injury. Data are presented as mean ± SEM. n=8 mice for control group, n=6 mice for other groups from one experiment, one-way ANOVA-Bonferroni post hoc. Source data are provided as a source data file.

Supplementary Figure S8. Additional analysis of NG2^{CreER}:MAPT^{mGFP} mice following difluorosamine treatment.

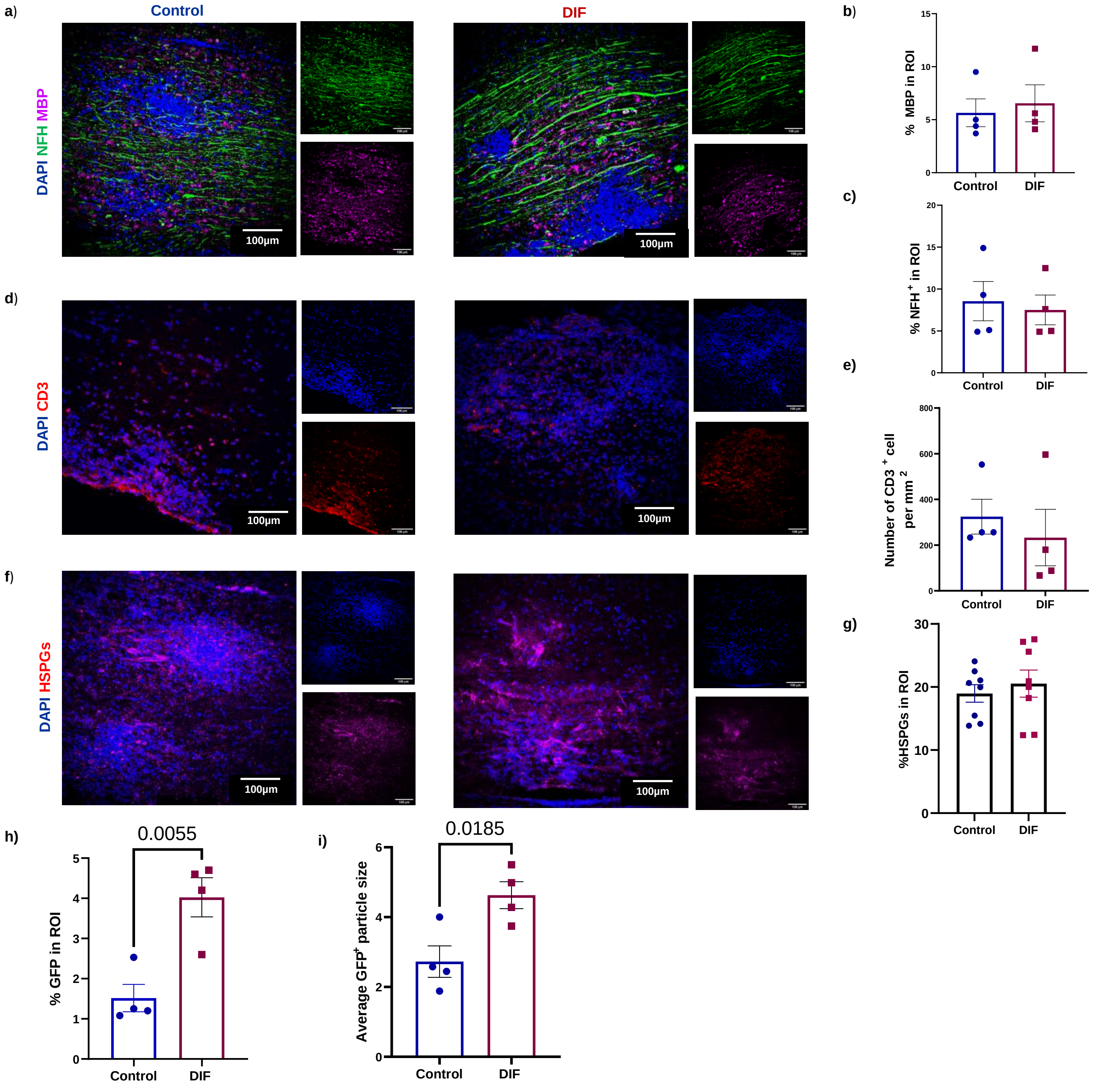

Spinal cords of NG2^{CreER}:MAPT^{mGFP} mice following DIF or vehicle treatment (Fig.7E) were immunostained for MBP (myelin), NFH (axons), CD3 (T cells) and HSPGs. High magnification immunofluorescence images acquired by laser confocal miscroscopy (Z-stack) (A, D, F) with bar graphs showing the percent of MBP; n=4 mice from one representative experiment that was repeated twice (B), NFH; n=4 mice from one representative experiment that was repeated twice (C), number of CD3^{+} T cells; n=4 mice from one representative experiment that was repeated twice (E) and percent of HSPGs; n=8 mice per group, data pooled from two independent experiments of 4 mice in each group) (G) in the lesion ROI defined by hypercellularity between DIF and vehicle treated mice. Data are presented as mean ± SEM. Two-tailed unpaired Student's t test. H-I) Bar graphs comparing the extent of GFP^{+} in region of interest (ROI) (H), and average particle size of GFP (I) in each mouse between DIF and vehicle treated mice. Data are presented as mean ± SEM from one representative experiment; n=4 mice in each group, each dot represents one mouse where a mean of 8 lesions were analyzed per mouse. This data was reproduced in a second experiment (source data file). Two-tailed unpaired Student's t test. Source data are provided as a source data file.
