# Supplementary material for: Versican promotes T helper 17 cytotoxic inflammation and impedes oligodendrocyte precursor cell remyelination
Source: Nat Commun. 2022 May 4;13:2445. doi: 10.1038/s41467-022-30032-0 (PMC9068758; doi:10.1038/s41467-022-30032-0)
Supplement: Supplementary file 2 — Reporting Summary [file 41467_2022_30032_MOESM2_ESM.pdf]

nature portfolio

Corresponding author(s): V. Wee Yong

Last updated by author(s): Mar 18, 2022

Reporting Summary

Nature Portfolio wishes to improve the reproducibility of the work that we publish. This form provides structure for consistency and transparency in reporting. For further information on Nature Portfolio policies, see our Editorial Policies and the Editorial Policy Checklist.

Statistics

For all statistical analyses, confirm that the following items are present in the figure legend, table legend, main text, or Methods section.

n/a	Confirmed	
☐	☒	The exact sample size (*n*) for each experimental group/condition, given as a discrete number and unit of measurement
☐	☒	A statement on whether measurements were taken from distinct samples or whether the same sample was measured repeatedly
☐	☒	The statistical test(s) used AND whether they are one- or two-sided<br>*Only common tests should be described solely by name; describe more complex techniques in the Methods section.*
☐	☒	A description of all covariates tested
☐	☒	A description of any assumptions or corrections, such as tests of normality and adjustment for multiple comparisons
☐	☒	A full description of the statistical parameters including central tendency (e.g. means) or other basic estimates (e.g. regression coefficient) AND variation (e.g. standard deviation) or associated estimates of uncertainty (e.g. confidence intervals)
☐	☒	For null hypothesis testing, the test statistic (e.g. *F*, *t*, *r*) with confidence intervals, effect sizes, degrees of freedom and *P* value noted<br>*Give P values as exact values whenever suitable.*
☒	☐	For Bayesian analysis, information on the choice of priors and Markov chain Monte Carlo settings
☒	☐	For hierarchical and complex designs, identification of the appropriate level for tests and full reporting of outcomes
☐	☒	Estimates of effect sizes (e.g. Cohen's *d*, Pearson's *r*), indicating how they were calculated

Our web collection on statistics for biologists contains articles on many of the points above.

Software and code

Policy information about availability of computer code

Data collection	Leica Application Suite X was used for confocal image acquisition. Olympus VS110 Slide scanner was used for both bright field and immunofluorescence microscopy.<br>Attune NxT Flow Cytometer (ThermoFisher) was used for quantifying T cell and dendritic cell populations.<br>QuantStudio 6 Flex Real-Time PCR System (Thermo Fisher) was used for gene expression analysis.<br>MetaXpress High-Content Image Acquisition and Analysis Software was used to acquire images for oligodendrocyte precursor cell culture experiments.<br>NovaSeq 6000 System (Illumina) was used for spatial single cell RNA sequencing.
Data analysis	ImageJ (NIH) 1.53c was used for confocal image analysis.<br>IMARIS 9.2.1 was used for 3D reconstruction of confocal z-stack images.<br>Attune NxT software (v3.1.2) was used for low cytometry experiments.<br>FlowJo v10 software was used for flow cytometry analysis.<br>MetaXpress High-Content Image Acquisition and Analysis Software was used for quantifying oligodendrocyte precursor cells.<br>Space Ranger software v.1.2, and STAR v.2.5.1 was used for genome alignment against the human reference dataset.<br>Loupe Browser 5.01 (10X Genomics) was used to visualize Spatial gene expression data from human MS tissue.<br>Microsoft Excel (Version 2201 Build 16.0.14827.20198) was used for collating data, and Graphpad Prism 9.0.2 was used for statistical analysis and generation of plots.

For manuscripts utilizing custom algorithms or software that are central to the research but not yet described in published literature, software must be made available to editors and reviewers. We strongly encourage code deposition in a community repository (e.g. GitHub). See the Nature Portfolio guidelines for submitting code & software for further information.

Data

Policy information about availability of data

All manuscripts must include a data availability statement. This statement should provide the following information, where applicable:

- Accession codes, unique identifiers, or web links for publicly available datasets
- A description of any restrictions on data availability
- For clinical datasets or third party data, please ensure that the statement adheres to our policy

The datasets generated and/or analyzed during the current study are available from the corresponding author on reasonable request.
Raw spatial RNA sequencing data are available at the NCBI Sequence Read Archive with the BioProject accession number: PRJNA734097
https://www.ncbi.nlm.nih.gov/bioproject/PRJNA734097

Field-specific reporting

Please select the one below that is the best fit for your research. If you are not sure, read the appropriate sections before making your selection.

[x] Life sciences [ ] Behavioural & social sciences [ ] Ecological, evolutionary & environmental sciences

For a reference copy of the document with all sections, see nature.com/documents/nr-reporting-summary-flat.pdf

Life sciences study design

All studies must disclose on these points even when the disclosure is negative.

Sample size	No sample size calculation was performed. Sample size was determined based on previously published results (Ref 16,19, 27), feasibility of the experiment, as well as the availability of sex and aged matched mice from transgenic mice.
Data exclusions	A few mice in EAE experiments were excluded due to death/euthanization prior to experimental endpoint because of EAE severity.
Replication	Findings were replicated and reproduced in multiple independent experiments. The number of independent experiments and replicates used are stated in the figures of the manuscript. All experimental findings were reproducible.
Randomization	All samples for experiments were randomized except for DIF treatment experiments. To have the similar mean clinical scores in both control and DIF groups, EAE mice were distributed between groups before starting the treatment according to their score .
Blinding	Investigators were blinded to group allocation during EAE scoring. Blinding was not relevant for other experiments/analyses since equal experimental parameters were consistently applied for each sample during data acquisition and data analysis.

Reporting for specific materials, systems and methods

We require information from authors about some types of materials, experimental systems and methods used in many studies. Here, indicate whether each material, system or method listed is relevant to your study. If you are not sure if a list item applies to your research, read the appropriate section before selecting a response.

Materials & experimental systems

n/a	Involved in the study	
[ ]	[x]	Antibodies
[x]	[ ]	Eukaryotic cell lines
[x]	[ ]	Palaeontology and archaeology
[ ]	[x]	Animals and other organisms
[ ]	[x]	Human research participants
[x]	[ ]	Clinical data
[x]	[ ]	Dual use research of concern

Methods

n/a	Involved in the study	
[x]	[ ]	ChIP-seq
[ ]	[x]	Flow cytometry
[x]	[ ]	MRI-based neuroimaging

Antibodies

Antibodies used	anti-versican V0/V1 (Millipore, ab1033, 1:100), anti-versican V0/V2 antibody (Millipore, ab1032, 1:100), anti-aggrecan (Millipore, ab1031, 1:100), anti-heparan sulfate proteoglycan (Amsbio, 370255,clone F58-10E4, 1:100), anti-fibronectin (Abcam, ab23750, 1:100), anti-fibrinogen (Abcam, ab34269, 1:100), anti-thrombospondin-1 (Abcam, ab85762, 1:100), anti-CSPG (Millipore, MAB2030, clone BE-123, 1:100), anti-myelin basic protein (MBP, Abcam, ab7349, 1:100), anti-Olig2 (Millipore, ab9610, 1:200), anti-platelet-derived growth factor receptor α (PDGFRα, R&D Systems, AF1062, 1:100), anti-adenomatous polyposis coli (APC, Millipore, clone CC-1, OP80, 1:100), anti-GFP (Aveslab, GFP-1020, 1:500), anti-ionized calcium-binding adaptor molecule1 (Iba1, Wako, 019-19741, 1:500), anti-CD3 (Abcam, ab5690,1:100), anti-mouse CD45 (BD Pharmingen, 550539, 1:50), anti-humanCD45 (Invitrogen,

MA5-17687, clone YAML501.4, 1:200), anti-human Versican V1 (Novus biologicals, NBP1-85432, 1:100), anti-BCAS1 (abcam, ab106661, 1:50), anti-oligodendrocyte marker O4 (R&D, MAB1326,IgM clone#4, 1:50),
APC anti-mouse IFN-γ (clone XMG1.2, Biolegend, 505810, 1:100), PE anti-mouse IL-17 (clone TC11-18H10.1, Biolegend, 506904, 1:100), and FITC anti-mouse/human FOXP3 (clone FJK-16s, eBioscience, 11-5773-82, 1:100),BV421 anti-mouse RORγt (clone Q31-378, BD Horizon, 1:100), Alexa Fluor 700 anti-mouse CD4 (clone RM4-5, BD Pharmingen, 557956, 1:100), PerCP/Cyanine5.5 anti-mouse CD8a (clone 53-6.7, Biolegend, 100733, 1:100) and APC-eFluor 780 anti-mouse CD3 Monoclonal (clone17A2, eBioscience, 47-0032-82, 1:100), PerCP anti-mouse/human CD44 (clone IM7, Biolegend, 103036, 1:100), Brilliant Violet 510 anti-mouse CD62L (clone MEL-14, Biolegend,104441, 1:100), APC-eFluor 780 anti-mouse CD25 (clone lPC61.5, eBioscience, 47-0251-82, 1:100), anti-mouse CD11b (clone M1/70, eBioscience, 14-0112-82, 1:100), APC anti-mouse CD80 (clone 16-10A1, Biolegend, 104714, 1:100), PE anti-mouse CD86 (clone GL-1, Biolegend, 105007, 1:100), PE/Cyanine7 anti-mouse CD11c (clone N418, Biolegend, 117318, 1:100), Alexa Fluor 700 anti-mouse F4/80 (clone BM8, Biolegend, 123130, 1:100), FITC anti-mouse I-A/I-E (clone M5/114.15.2, Biolegend, 107605, 1:100), PerCP/Cyanine5.5 anti-mouse CD317 (BST2, PDCA-1) (clone 927, Biolegend, 127022, 1:100), APC 750 anti-mouse CD24(clone M1/69, Biolegend, 101839, 1:100), V450 anti-mouse IL-12 (p40/p70) (clone C15.6, BD Horizon,561456, 1:100).
The follow secondary antibodies from Jackson ImmunoResearch were used at 1:400 dilution: Alexa Fluor 488 donkey anti-mouse IgM (Jackson ImmunoResearch 715-545-020); Alexa Fluor 647 donkey anti-mouse IgM (Jackson ImmunoResearch 715-605-020); Alexa Fluor 488 donkey anti-mouse IgG (Jackson ImmunoResearch 715-545-150); Alexa Fluor 488 donkey anti-chicken IgY (Jackson ImmunoResearch 703-545-155); Cyanine Cy3 donkey anti-rat IgG (Jackson ImmunoResearch 712-165-150); Alexa Fluor 488 donkey anti-goat IgG (Jackson ImmunoResearch 705-546-147), Alexa Fluor 488 donkey anti-rabbit IgG (Jackson ImmunoResearch 711-545-152); and Alexa Fluor 647 donkey anti-rabbit IgG (Jackson ImmunoResearch 711-605-152).

Validation

All antibodies are from commercial sources and have been validated by the manufactures. Detailed information can be found at the following links:

Abcam
https://www.abcam.com/primary-antibodies/how-we-validate-our-antibodies
IHC and ICC determine whether an antibody recognizes the correct protein based on cellular and subcellular localization. Antibody specificity is confirmed by looking at cells that either do or do not express the target protein within the same tissue. Initially, our scientists will review the available literature to determine the best cell lines and tissues to use for validation. We then check the protein expression by IHC/ICC to see if it has the expected cellular localization . If the localization of the signal is as expected, this antibody will pass and is considered suitable for use in IHC/ICC. We use a variety of methods, including staining multi-normal human tissue microarrays (TMAs), multi-tumor human TMAs, and rat or mouse TMAs during antibody development. These high-throughput arrays allow us to check many tissues at the same time, providing uniformly as all tissues are exposed to the exact same conditions. To ensure the same, accurate results can be obtained across batches of the same antibody, we perform consistency tests to assess batch-to-batch variation. In the case of recombinant antibodies, consistency between batches is very high, meaning you are unlikely to need to perform additional optimization procedures (eg titration experiments) between batches. This may not be the case with other non-recombinant hybridoma-produced monoclonal and polyclonal antibodies where the degree of variation and drift is inherently higher. When available and suitable for assay development, recombinant monoclonal antibodies are favored and provide the best batch-to-batch consistency. We are currently working towards using KO cell lines for our ICC validation.

Amsbio
https://www.amsbio.com/ultramab-antibodies/
Performance and specificity are the pre-requisites for antibodies to be used for diagnostic and therapeutic applications. AMSBIO supplies UltraMAB®, a unique line of ultraspecific and extensively validated monoclonal antibodies, to ensure superior performance in these areas. A high density protein microarray chip has been developed for antibody specificity testing, and has been applied to identify UltraMAB® antibodies.

Aveslab
https://www.aveslabs.com/pages/custom-chicken-antibody-production
Aves antibodies come either as an "IgY fraction" which is >90% pure antibody or as an “affinity purified antibody,” which is 100% pure antibody. The IgY fraction product contains all of the antibody circulating in the hen at the time of egg production, while the affinity purified product contains only those which recognize and bind with high affinity to the antigen molecule.

BioLegend
https://www.biolegend.com/en-us/reproducibility
All newly developed clones at BioLegend undergo validation testing for multiple applications. This serves as a cross-check for specificity and provides clarity for research uses. Typically, antibodies are tested by two or more of the below methods. Knockout or knockdown of gene expression, such as with siRNA, is also an excellent tool for target validation. BioLegend antibodies undergo an extensive series of testing to ensure quality at every step in the manufacturing process, as well as maintaining quality after the sale.

Millipore
https://www.emdmillipore.com/CA/en/life-science-research/antibodies-assays/antibodies-overview/Antibody-Development-and-Validation
These efforts and collaborations have led to new validation techniques and novel antibody-based technologies, such as improved bead-based multiplex assays and imaging flow cytometry.

Novus
https://www.novusbio.com/reproducibility.html
Novus recognizes the need for highly validated, high quality antibodies in the life sciences community. The research community faces ongoing concerns about data reproducibility and especially the validity of antibody-based assays. A recent article in Nature discusses the variable standards and performance of antibodies and antibody suppliers in the market. Novus is committed to addressing this

problem and to helping our customers attain the best possible results with our products.
To that end, we actively seek high quality, highly validated products and provide support to ensure that our customers have the tools to properly validate their own assays. We are also collaborating with several global initiatives that help life science researchers choose antibodies with proven results. Of the five pillars of validation established by these initiatives, genetic knockout validation provides the most reliable control for assessing antibody specificity. For technical support in validating your antibody based applications, visit technical support.

R&D Systems
https://www.rndsystems.com/quality/antibodies-built-for-reproducibility
With the recent reports stating antibodies as one of the reasons for scientific irreproducibility, you can rest assured with our antibodies. R&D Systems® takes rigorous steps towards antibody validation and reproducibility. We have been since the beginning. For 30 years, we have used our industry-leading production standards and quality control specifications to develop antibodies that can be relied on for specificity and reproducibility. By developing and testing our products in-house, we can ensure a validated and specific antibody. We are confident in our antibodies and provide 100% guarantee for our products.

ThermoFisher/ Invitrogen /eBioscience/BD
https://www.thermofisher.com/ca/en/home/life-science/antibodies/invitrogen-antibody-validation.html
Part 1—Target specificity verification:This helps ensure the antibody will bind to the correct target. Our antibodies are being tested using at least 1 of the following methods to ensure proper functionality in researcher’s experiments. Click on each testing method below for detailed testing strategies, workflow examples and data figure legends.
Knockout—expression testing using CRISPR-Cas9 cell models
Knockdown—expression testing using RNAi to knockdown gene of interest
Independent antibody verification (IAV)—measurement of target expression is performed using two differentially raised antibodies recognizing the same protein target
Cell treatment—detecting downstream events following cell treatment
Relative expression—using naturally occurring variable expression to confirm specificity
Neutralization—functional blocking of protein activity by antibody binding
Peptide array—using arrays to test reactivity against known protein modifications
SNAP-ChIP™—using SNAP ChIP to test reactivity against known protein modifications
Immunoprecipitation-Mass Spectrometry (IP-MS)—testing using immunoprecipitation followed by mass spectrometry to identify antibody targets
Part 2—Functional application validation
These tests help ensure the antibody works in a particular application(s) of interest, which may include (but are not limited to):
Western blotting
Flow cytometry
ChIP
Immunofluorescence imaging
Immunohistochemistry
Most antibodies were developed with specific applications in mind. Testing that an antibody generates acceptable results in a specific application is the second part of confirming antibody performance.

Advanced Verification
Thermo Fisher Scientific is committed to adopting validation standards for our Invitrogen antibody portfolio. The Advanced Verification badge is applied to products that have passed application and specificity testing. This badge can be found in the search results and at the top of the product specific web pages. Data supporting the Advanced Verification badges can be found in product specific data galleries.

Animals and other organisms

Policy information about studies involving animals; ARRIVE guidelines recommended for reporting animal research

Laboratory animals	The following stains of mice were used for this study: Female mice were used between 8 to 10 weeks of age.Female C57Bl/6J mice (6-8 weeks old) and litters from pregnant CD1 mice (P1-P2) were purchased from Charles River and used for in vitro leukocyte or OPC cultures, respectively. NG2CreER (JAX 008538) mice and TaumGFP (JAX 021162) mice aged 6 to 8 weeks were acquired from Jackson Laboratories and bred in University of Calgary Animal facility to produce female NG2CreER:MAPTmGFP mice (8-10 week old for EAE experiments or lysolecithin demyelination); the 2D2 TCR (TCRMOG) transgenic mice (JAX 006912) were also from Jackson Laboratories. Mice were housed between 21 and 23 degrees Celcius, in low humidity, with 12hrs. light and 12hrs. dark cycle from 7am light and starting 7pm dark.
Wild animals	No wild animals were used for this study.
Field-collected samples	No field-collected samples were used for this study.
Ethics oversight	All experiments were performed with ethics approval (protocol number AC21-0174) from the Animal Care Committee at the University of Calgary under regulations of the Canadian Council of Animal Care.

Note that full information on the approval of the study protocol must also be provided in the manuscript.

Human research participants

Policy information about studies involving human research participants

Population characteristics	From the UK Multiple Sclerosis Tissue Bank:<br>45 years old female (MS163), RRMS, disease duration 6 years<br>42 years old female (MS230), SPMS, disease duration 19 years<br>43 years old male (MS352), SPMS, disease duration 16 years<br><br>From CRCHUM:<br>60 years old female (MS 172), SPMS<br>61 years old male (MS200), SPMS<br><br>From Netherlands Brain Bank:<br>58 years old male (12-078), SPMS, disease duration 30 years<br>53 years old female (13-019), RRMS, disease duration 16 years<br>48 years old female (13-047), PPMS, disease duration 22 years<br>51 years old female (14-007), PPMS, disease duration 23 years<br>35 years old female (14-038), SPMS, disease duration 10 years<br>57 years old female (15-006), SPMS, disease duration 28 years
Recruitment	Postmortem frozen brain tissues from MS patients with progressive disease course were obtained from the UK Multiple Sclerosis Tissue Bank at Imperial College and from MS patients with full ethical approval (BH07.001) and informed consent as approved by the CRCHUM research ethics committee.<br>Paraffin-embedded sections from autopsied MS subjects were obtained from the Netherlands Brain Bank (https://www.brainbank.nl), Amsterdam. All samples were collected with full informed consent for autopsy, and their use for research has been approved by local institutional ethics committee.
Ethics oversight	Postmortem frozen brain tissues from people with MS patients and healthy control brain tissue were obtained from the UK Multiple Sclerosis Tissue Bank at Imperial College, London (www.ukmstissuebank.imperial.ac.uk; kindly provided by Dr. Djordje Gveric). Secondary progressive MS tissues used for spRNAseq were obtained from Dr. Alex Prat, University of Montreal. with full ethical approval (BH07.001, Nagano 20.332 - YP) and informed consent as approved by the CRCHUM and University of Montreal research ethics committee. Paraffin-embedded sections from autopsied MS subjects were from the Netherlands Brain Bank (https://www.brainbank.nl/), Amsterdam. All samples were collected with full informed consent for autopsy.

Note that full information on the approval of the study protocol must also be provided in the manuscript.

Flow Cytometry

Plots

Confirm that:

- [x] The axis labels state the marker and fluorochrome used (e.g. CD4-FITC).
- [x] The axis scales are clearly visible. Include numbers along axes only for bottom left plot of group (a 'group' is an analysis of identical markers).
- [x] All plots are contour plots with outliers or pseudocolor plots.
- [x] A numerical value for number of cells or percentage (with statistics) is provided.

Methodology

Sample preparation	After a quick PBS perfusion,the spinal cords from EAE mice were dissected, minced, and enzymatically dissociated by incubating with 2.5 mg trypsin and 5 mg collagenase in 5 ml DMEM media for 20 min at 37°C. To remove the myelin debris, cells were overlaid on Debris Removal Solution (Miltenyi Biotec) according to the manufacturer's instructions. An average of 2 million cells was isolated from each animal.<br>Lymph nodes were isolated, minced and filtered through a sterile cell strainer to obtain single-cell suspensions.<br>Naïve CD4+ T Cells were isolated from single-cell suspensions of splenocytes using the EasySep Kit (STEMCELL) by negative selection and then polarized to T helper subsets.<br>Bone marrow-derived dendritic cell (BMDC) culture was prepared from femurs and tibiae of C57/BL6 mice.
Instrument	Attune NxT Flow Cytometer (ThermoFisher)
Software	Attune NxT Software v 3.1 and FlowJo v10 software
Cell population abundance	Viability was ~60-80% and final sorted cells were >1 % of the viable population.

Gating strategy	First, forward and side scatter (FSC vs SSC) gating was used to filter cells based on size and granularity (complexity). Then, viable cells were selected. CD3+ and CD4+ cells were sent to next gates.

☒ Tick this box to confirm that a figure exemplifying the gating strategy is provided in the Supplementary Information.
